# Predicting mortality from change-over-time in the Charlson Comorbidity Index: A retrospective cohort study in a data-intensive UK health system

**DOI:** 10.1097/MD.0000000000004973

**Published:** 2016-10-28

**Authors:** Paolo Fraccaro, Evangelos Kontopantelis, Matthew Sperrin, Niels Peek, Christian Mallen, Philip Urban, Iain E. Buchan, Mamas A. Mamas

**Affiliations:** a Health eResearch Centre, Farr Institute for Health Informatics Research; b NIHR Greater Manchester Primary Care Patient Safety Translational Research Centre, Institute of Population Health; c NIHR School for Primary Care Research, University of Manchester, Manchester; d Research Institute for Primary Care & Health Sciences, Arthritis Research UK Primary Care Centre, Keele University, Keele, Staffordshire, United Kingdom; e Cardiovascular Department, Hôpital de La Tour, Geneva, Switzerland; f Keele Cardiovascular Research Group, Keele University Stoke-on-Trent and Royal Stoke Hospital, University Hospital North Midlands, Stoke-on-Trent, United Kingdom.

**Keywords:** Charlson Comorbidity Index, comorbidity, multimorbidity, prognostic impact, retrospective cohort study, risk stratification, Salford Integrated Record, survival analysis

## Abstract

Multimorbidity is common among older people and presents a major challenge to health systems worldwide. Metrics of multimorbidity are, however, crude: focusing on measuring comorbid conditions at single time-points rather than reflecting the longitudinal and additive nature of chronic conditions. In this paper, we explore longitudinal comorbidity metrics and their value in predicting mortality.

Using linked primary and secondary care data, we conducted a retrospective cohort study on adults in Salford, UK from 2005 to 2014 (n = 287,459). We measured multimorbidity with the Charlson Comorbidity Index (CCI) and quantified its changes in various time windows. We used survival models to assess the relationship between CCI changes and mortality, controlling for gender, age, baseline CCI, and time-dependent CCI. Goodness-of-fit was assessed with the Akaike Information Criterion and discrimination with the c-statistic.

Overall, 15.9% patients experienced a change in CCI after 10 years, with a mortality rate of 19.8%. The model that included gender and time-dependent age, CCI, and CCI change across consecutive time windows had the best fit to the data but equivalent discrimination to the other time-dependent models. The absolute CCI score gave a constant hazard ratio (HR) of around 1.3 per unit increase, while CCI *change* afforded greater prognostic impact, particularly when it occurred in shorter time windows (maximum HR value for the 3-month time window, with 1.63 and 95% confidence interval 1.59–1.66).

Change over time in comorbidity is an important but overlooked predictor of mortality, which should be considered in research and care quality management.

## Abstract 2

Supplemental Digital Content is available in the text

## 1 Introduction

In “ageing” populations the prevalence of patients with multiple conditions increases^[^1 2^]^ placing extra demands on healthcare systems.^[^3 4^]^ Population-based studies have revealed the presence of at least one long-term condition in over a third of patients,^[^2 5^]^ with two-thirds of those aged over 65 years and three-quarters of those aged over 85 years having at least 2 concurring conditions.^[6]^

Linked electronic health records (EHRs) may offer new information about multimorbidity.^[7]^ Some EHRs hold comorbidity scores,^[8]^ ranging from simple summation of the number of conditions to more complex scores that assign different weights to diseases in respect of their prognoses.^[^9–13^]^ Although EHRs can provide rich longitudinal information most studies use the data available at a single time-point to measure comorbidity, which treats it as a static phenomenon when it is logically dynamic.^[^14–16^]^ Similarly in prognostic studies, only those comorbid conditions present at baseline are commonly considered, while new conditions arising may affect the outcome of interest.^[^14–16^]^ While it is reasonable to hypothesize that those with rising comorbidity over time may have worse health outcomes^[15]^ this group of patients are poorly characterized in the literature.

This study aimed to characterize the distribution, and changes over time, of comorbidities, as measured by the Charlson Comorbidity Index (CCI),^[13]^ in a UK population with high-quality EHRs. We also sought to investigate different ways to account for longitudinal patterns of comorbidity in survival analyses and see if this enhanced the prediction of mortality.

## 2 Methods

### 2.1 Data source

Data were extracted from the Salford Integrated Record (SIR)—an anonymized extract of linked data from all 53 primary care providers and 1 secondary care provider in the UK City of Salford (population in Census 2011 of ∼235k^[17]^). The data in SIR includes all primary care and secondary care records (i.e., focused on long-term conditions management) as well as all results from biochemical testing across primary and secondary care. Data are stored as Read codes v2 and v3.^[18]^

Salford is a relatively deprived area, with almost a third of neighborhoods in the most deprived tenth for England.^[19]^ In terms of multimorbidity burden, Salford is in the 61st centile, as measured by England's primary care Quality and Outcome Framework (QOF).^[20]^

### 2.2 Study period and population

The study period was from April 1, 2005 to December 31, 2014. As QOF has been proven to influence general practitioners data recording behaviors and improve data quality on included clinical conditions,^[^21–24^]^ we focused on the period after QOF was introduced and used its financial years (1 April to 31 March). We used an open cohort design and included all patients aged 18 years or older, registered in one of the SIR primary care practices. Patients were considered as participating in the study until death or migration out of the area.

### 2.3 Comorbidity burden measurement: Charlson Comorbidity Index calculation

We measured comorbidity burden by using the CCI^[13]^—a widely used score,^[8]^ which has different weights for 22 clinical conditions in relation to their impact on prognosis. Although originally developed to predict mortality risk after hospitalization, it has been shown to independently predict adverse outcomes across a broad spectrum of conditions.^[^25–35^]^

We calculated the CCI on the basis of the work of Khan et al,^[36]^ who provided a list of validated Read diagnostic codes for calculating it in UK primary care. Every time a relevant Read diagnostic code was found for a patient, the CCI was updated using the weights for the related disease category. Age was modeled separately and not included in the CCI calculation.

Because of privacy restrictions on access to data about sexual or mental health we were not able to include human immunodeficiency virus (HIV)/acquired immunodeficiency syndrome (AIDS) and dementia in our study.

In addition to the original CCI definition, we stratified the disease categories into cardiovascular (i.e., myocardial infarction, congestive heart failure, peripheral vascular disease, cerebrovascular disease, diabetes mellitus, renal disease) and noncardiovascular (i.e., peptic ulcer disease, cancer, metastatic disease, hemiplegia, liver disease, chronic pulmonary disease) diseases. We then repeated the process explained above and obtained 2 individual scores (cardiovascular CCI and noncardiovascular CCI).

### 2.4 Data analysis

To investigate the proportion of patients experiencing changes in comorbidities during follow-up, we calculated the difference between patient CCI values at baseline, then at 1, 5, and 10 years. For each follow-up period, we next calculated the overall proportion of patients that had a CCI change and their mortality rates. We repeated this analysis by stratifying for the CCI value at baseline (i.e., 0, 1, 2, ≥3) and reported separately proportion of change and crude mortality rate for CCI changes of 0, 1, 2, and ≥3.

To evaluate the prognostic importance of comorbidity burden changes over time and the time period over which changes occur, we performed survival analyses using Cox regression models^[37]^ with time to death from any cause as the outcome. We built three different datasets by discretizing time into 3-, 6-, and 12-month time windows (see Supplementary Figure 1 and Table 1) and implemented different models by increasing the level of model's complexity. The models considered:

1. Age, gender, and CCI at baseline (*model 1*).
2. Gender and time-dependent age and CCI (*model 2*).
3. Gender and CCI at baseline as well as time-dependent age and CCI (*model 3*).
4. Gender and baseline CCI value in addition to time-dependent age and cumulative CCI change from baseline (*model 4*).
5. Gender and time-dependent age, CCI and CCI change over consecutive time windows (*model 5*).

For both the nonstratified and cardiovascular stratified analyses, time-dependent covariates were modeled by updating their values at the beginning of each time window (see Supplementary Table 1).

We used the Akaike Information Criterion (AIC) to assess model goodness-of-fit.^[38]^ For each model, we also assessed discrimination with 95% confidence intervals (CIs) for the c-statistic by calculating c-index over 100 bootstrap iterations. Finally, we calculated models’ Variance Inflation Factors (VIF), which assesses collinearity between covariates, and checked the proportional hazards assumption.

### 2.5 Sensitivity analyses

We performed several sensitivity analyses. First, we evaluated possible clustering effects related to the different primary care practices from which the data arose by repeating our main analysis with the addition of a random intercept at practice level. Second, since the currency of the original CCI disease weights is under debate, we repeated all analyses with an updated version of the CCI.^[39]^ Third, we only considered the patients that experienced a change in CCI during the follow-up. Fourth, we repeated all analyses by categorizing both CCI and CCI change as 0, 1, 2, ≥3 and assessing interaction terms between CCI value and CCI change. Finally, as c-statistic to compare different prediction models has been criticized,^[^40 41^]^ we also compared the simplest (model 1) and most complex (model 5) of the models we tested in terms of Net Reclassification Improvement (NRI) and Integrated Discrimination Improvement (IDI)^[^40 41^]^ to quantify differences in predictive ability. We based our analysis on Wong et al^[42]^ who calculated IDI and NRI to compare a time-fixed and time-dependent model in a survival analysis. Particularly, we calculated IDI as the difference between the mean predicted risk in patients who died and patients who did not die for both models. As in the context of our analysis there are no clear risk categories to which patients are assigned, we implemented a category-less NRI and calculated the proportion of the correct (i.e., model 5 predicted higher risk than model 1 for patients who died) minus incorrect predictions in the events plus the proportions of correct (i.e., model 5 predicted lower risk than model 1 for patients who did not die) minus incorrect predictions for nonevents. For both IDI and NRI values above 0 indicate better performance. We calculated 95% CIs for both IDI and NRI for each time point over 100 bootstrap iterations.

## 3 Results

### 3.1 Study population characteristics

A total of 357,829 patients were recorded in the SIR database during the study period. We excluded 65,182 patients because they were under the age of 18 years and 5188 patients because of conflicting registration data (such as temporary residents). A total of 287,459 patients were included in the analysis, with a mortality rate of 5.7% (N = 16,452) recorded during the study period. Table 1 shows patient characteristics at baseline. The proportion of women was 49.3% and mean age at baseline was 38.3 years (standard deviation [SD] 18.8), with a mean follow-up time of 7.9 years (SD 2.8). Mean deprivation as measured by the Townsend score^[43]^ which incorporates four variables (i.e., unemployment, noncar ownership, nonhome ownership, and household overcrowding) to calculate material deprivation within a population, was 1.9 (SD 3.4). The majority of patients were Caucasian (85.8%). Mean body mass index (BMI) at baseline was 25.8 kg/m^2^ (SD 5.7). The prevalence of CCI disease categories at baseline varied from 0.1% to 13.2%, with chronic pulmonary disease having the highest prevalence, followed by diabetes (3.5%). Prevalence rates for cancer, cerebrovascular disease, myocardial infarction, peptic ulcer disease, and musculoskeletal disease varied between 1% and 2%, whilst for all other comorbidities prevalence rates were below 1%.

**Table 1:**
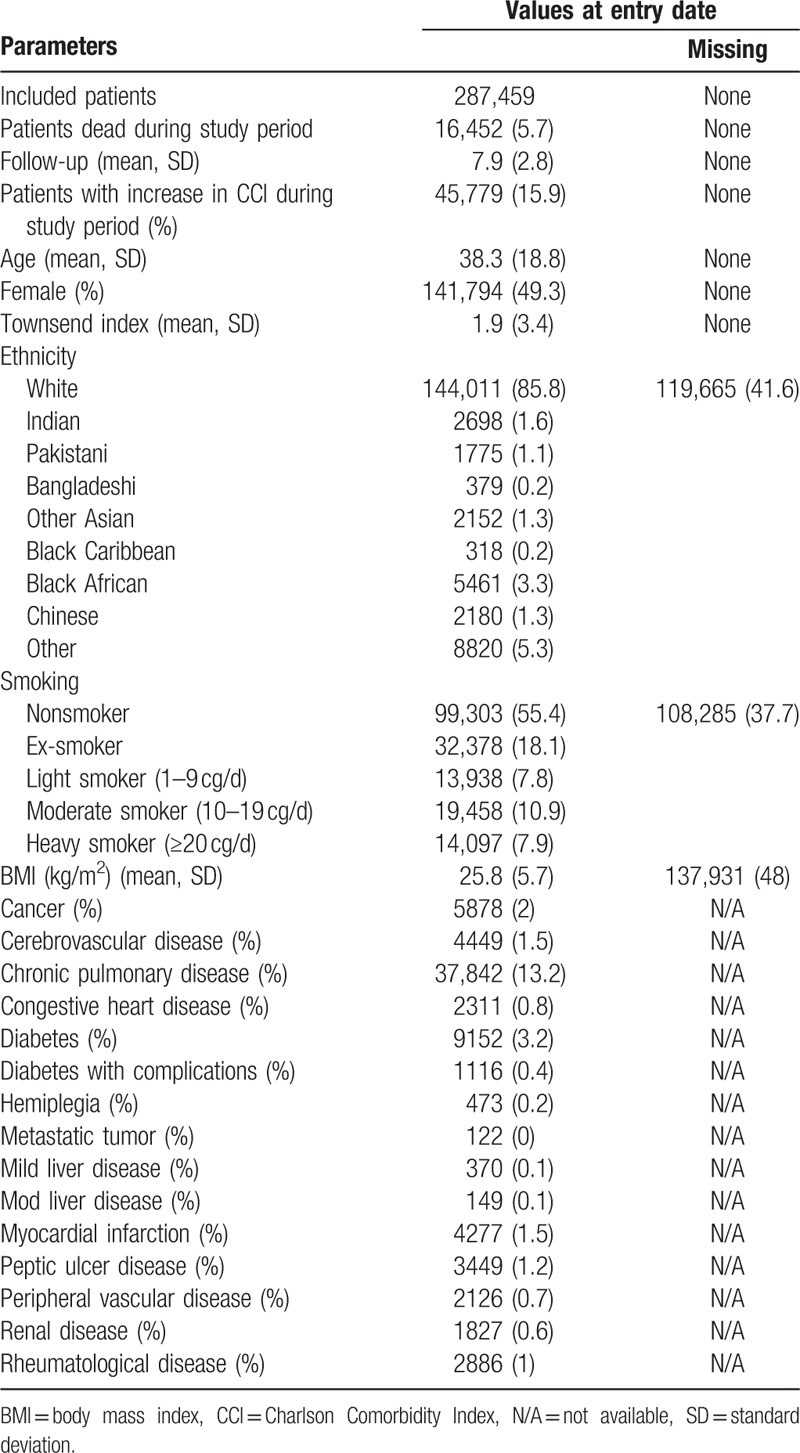
Patient characteristics at baseline.

Table 2 reports trends over time of prevalence of the CCI diseases categories (see Supplementary Figure 2 for graphical representation). Prevalence rates for cancer, chronic pulmonary disease, and diabetes increased during the study period, while they decreased for myocardial infarction, peptic ulcer, and musculoskeletal disease. Renal disease prevalence peaked in financial years 2009/10 and 2010/11 and then slightly decreased. All the other disease categories remained stable.

**Table 2:**
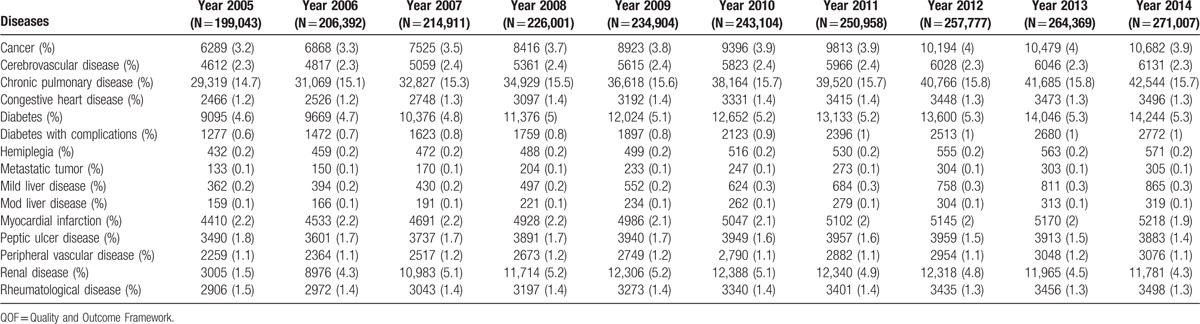
Charlson Comorbidity Index disease categories trend over the study period (on QOF financial years, such as 1st of April to 31st March of the next year) in terms of number of patients affected and prevalence.

### 3.2 Comorbidities change and mortality

Over the study period, we observed a change in CCI at 1 year for 5533 (1.9%) patients, with a crude mortality rate documented within this group of 3.1%. The number of patients for whom we observed CCI changes after 5 and 10 years were 30,025 (10.4%) and 45,096 (15.9%), with a respective crude mortality rate of 10.0% and 19.8%. When comparing mortality between the group of patients that had a change in CCI and those that did not we found odds ratios of 8.8 (95% CI: 7.5–10.4), 6.6 (95% CI: 6.3–6.9), and 7.8 (95% CI: 7.5–8.0) at the 3 time points, respectively.

Table 3 reports the mortality odds ratios associated with a change in CCI of 1, 2, and equal or more than 3 units, respectively (see Supplementary Figures 3–5 for details about prevalence of CCI change and related mortality). Overall, the odds ratios increased for bigger CCI changes and decreased for longer follow-up times and higher baseline CCI values. All comparisons were statistically significant (*P* values lower than 0.05), with the exception of some analyses for baseline CCI 2 and 3.

**Table 3:**
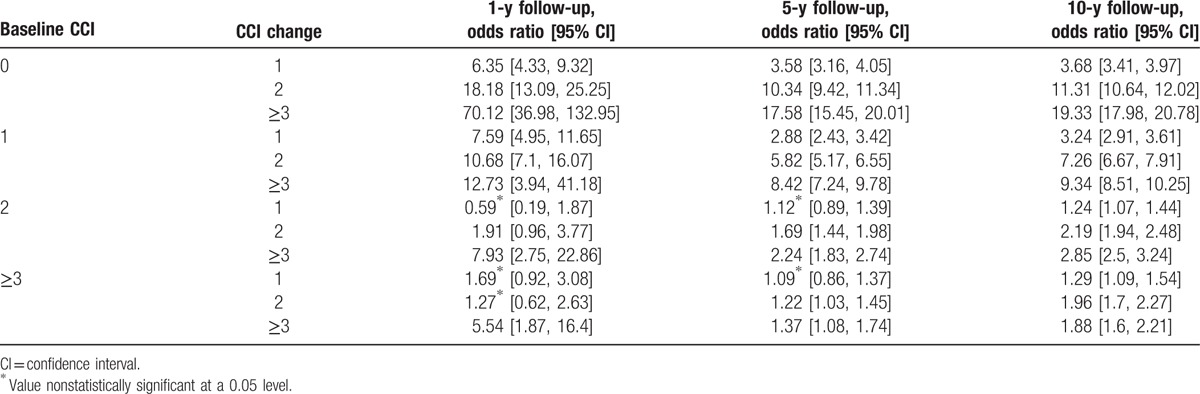
Odds ratio of mortality for group of patients that had a change in Charlson Comorbidity Index (CCI) and the patients that did not have it for different baseline CCI values across the study.

### 3.3 Regression analyses

Table 4 summarizes covariates prognostic impact (per unit increase), AIC, and c-statistic for the 6-month time window analysis. These are reported separately for the nonstratified and cardiovascular-stratified analyses.

**Table 4:**
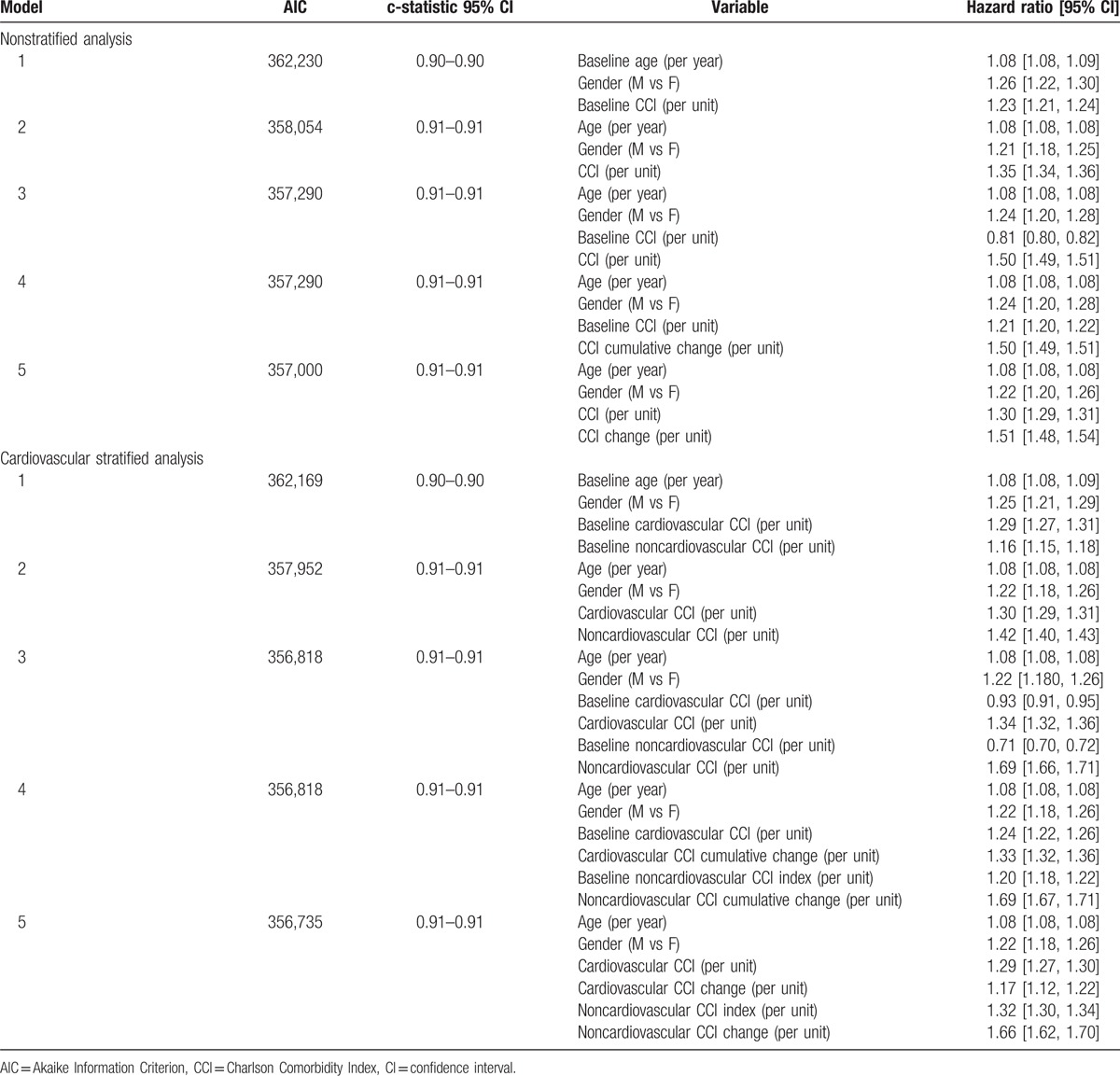
Results for the 6-month time windows in terms of AIC and hazard ratios.

We observed the same prognostic impact (HR 1.50, 95% CI: 1.49–1.51) in model 3 for the time-dependent CCI value and model 4 for the CCI cumulative change over study period, which in both cases was much greater than the baseline CCI value (HR 0.81, 95% CI: 0.80–0.82 in model 3 and HR 1.21, 95% CI: 1.20–1.22 in model 4). In addition, it can be seen that longitudinal changes in CCI provide additional prognostic information (HR 1.51, 95% CI: 1.48–1.54) to that provided by the absolute CCI score (HR 1.30, 95% CI: 1.29–1.31) also when looking at changes across different time windows (i.e., model 5). Interestingly, longitudinal changes in the noncardiovascular components of CCI provide a much greater hazard for mortality (HR 1.66, 95% CI: 1.62–1.70) than the cardiovascular components (HR 1.17, 95% CI: 1.12–1.22).

Increasing the model complexity from model 1 to model 5 led to a better fit of the models to the data, as witnessed by a decrease in AIC, but not a substantial improvement in the c-statistic. Model 3 and model 4 were equivalent in terms of goodness-of-fit (i.e., same AIC value). VIF values were lower than 2 for all included variables across all models, showing no indication of collinearity between the covariates. Finally, there was no evidence to reject the hypothesis of proportional hazards.

Results from the analyses with 3- and 12-month time windows showed similar findings to those undertaken with 6-month time windows.

Table 5 shows hazard ratios for model 5, such as the model that obtained the best AIC values, across all the three different time windows (i.e., 12-, 6-, and 3-month time windows). Looking at the nonstratified analysis, CCI (per unit increase) had similar prognostic impact across all analyses, whilst longitudinal changes in CCI hazard ratio (per unit increase) augmented with shorter time windows. The cardiovascular-stratified analysis showed similar figures, whilst the noncardiovascular CCI score had bigger prognostic impact in shorter time periods.

**Table 5:**
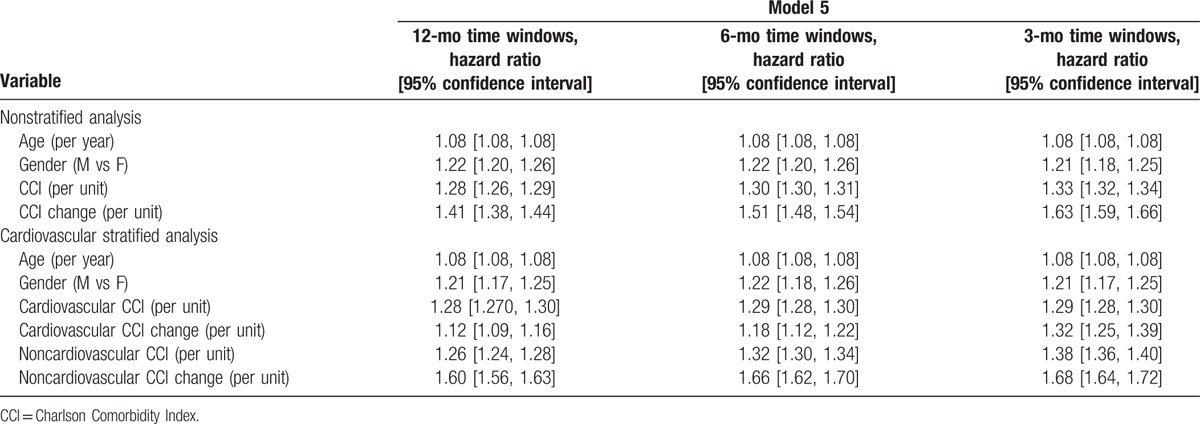
Hazard ratios for model 5 across the different time windows analyses.

The sensitivity analysis that focused only on patients that experienced CCI changes gave comparable results to the main analysis, with the exception of an increased difference in c-statistic, for which we observed values for the 95% CI ranging from 0.79 to 0.79 for model 1 to 0.83 to 0.84 for model 5 in the 6-month time window analysis. The sensitivity analysis that compared model 1 and 5 in terms of IDI and NRI showed that for most time points the 2 models had the same predictive ability (see Supplementary Figure 6), with model 5 that was slightly better in IDI and model 1 in NRI. Sensitivity analyses, exploring variations by care provider, disease weighting schemes and score categorizations, showed similar results to our main analysis.

## 4 Discussion

Our population-based analysis in over a quarter of a million people with detailed primary care records suggests that comorbidity is a dynamic process, with 1 in 10 patients showing a change in CCI over 5 years. This longitudinal pattern of comorbidity was associated with increased mortality risk, where change over time in CCI was a stronger predictor than CCI at baseline. In addition, the more rapid changes in CCI posed a greater mortality risk.

This study confirms that as populations “age,” a significant proportion of patients will experience comorbidity changes over time, and that longitudinal uses of metrics like CCI hold important prognostic information. Specifically, using Cox regression models with time-dependent CCI and including CCI-change provides additional prognostic information that should be considered when studying long-term outcomes in EHRs.

Regarding the choice of variables and how they are included in a regression model, we did not observe much variation in terms of discriminatory ability, with models 2 to 5 being almost equivalent. To increase the contrasts between models we restricted the discrimination comparison to the 15.9% of the population with comorbidity changes, however, the differences did not greatly increase. Similar predictive performance between models was also found in the IDI and NRI sensitivity analysis.

Since many of the comorbidities comprising the CCI score are heterogeneous, we stratified our analysis by 2 broad condition groups: with separate cardiovascular and noncardiovascular variables. In predicting mortality: for the *cardiovascular CCI*, absolute score had a greater impact than its change over time, whilst for the *noncardiovascular CCI* change over time was more predictive than absolute value. A possible explanation is that cardiovascular diseases are yoked in common pathophysiological mechanisms with similar progression over time and share overlapping treatment strategies, hence longitudinal changes in the burden of these conditions is less likely to be as important when one already has an existing cardiovascular disorder. In contrast, noncardiovascular CCI encompasses a heterogeneous group of diseases such as cancer, peptic ulcers, pulmonary pathology, or liver diseases with separate pathophysiological mechanisms and treatment strategies whose prognostic impact is likely to be additive hence why dynamic changes in noncardiovascular CCI has such important prognostic implications.

Although multimorbidity is often considered a static process,^[^14–16^]^ studies have analyzed 3 main aspects of longitudinal changes in comorbidities^[^14–16 44–46^]^: finding trajectories of comorbidity evolution over time^[^16 44 46^]^; investigating the best way of longitudinally modeling comorbidities when making predictions^[^14 15^]^; and assessing if prognostic impact of comorbidities is temporary or persistent.^[45]^ Comorbidities were mostly defined as counts of diseases,^[^16 44–46^]^ with CCI used in just 2 studies^[^14 15^]^ and no comparisons made between the cardiovascular and noncardiovascular components of CCI. Whilst CCI “weights” clinical conditions by their prognostic impact, a simple count of comorbidities would be confounded by the fact that different clinical conditions will impact on prognosis differently. Therefore, a change in the number of comorbid conditions will have very different prognostic implications depending on which conditions have changed.

To date, the studies of Aarts et al^[45]^ and Strauss et al^[16]^ are the only ones to report the number of patients experiencing change in comorbidities over time. Aarts et al^[45]^ followed 1184 patients aged 24 to 81 for 6 years in a Dutch prospective study and reported that 16.4% with changes in comorbidities. Strauss et al^[16]^ studied 24,641 people aged >50 for 3 years in a UK primary care setting and reported 60% of these older patients had changes in comorbidities. Our study was most comparable with that of Aarts et al^[45]^ as we considered similar age groups, and our results for overall change over time in comorbidity were very similar.

Four studies have related advancing multimorbidity to worse health outcomes,^[^14–16 45^]^ and we extended the methodologies used. Aarts et al^[45]^ and Strauss et al,^[16]^ used latent class analysis to identify different multimorbid trajectories in primary care data, and found worse self-reported health among patients with greater (especially steeper) changes in comorbidities. Zeng et al^[15]^ associated steeper CCI yearly change with worse general health in older (>65 years) patients with at least 3 comorbidities (N = ∼15,000) over a 10-year period. Finally, Wang et al^[14]^ reported much greater prognostic impact for time-dependent CCI levels compared to CCI values at baseline amongst a population of United States Medicare patients older than 65 years (N = 50,000). Our model 3 reflected that of Wang et al and we found it fitted less well than the model that explicitly considered CCI changes over different time windows, and identified baseline CCI value as a protective factor, which is counterintuitive. These results suggest that the explicit inclusion of CCI changes allowed us to better capture and describe the complexity of comorbidity burden evolution over time.

Strauss et al,^[16]^ Lappenschaar et al,^[44]^ and Quiñones et al^[46]^ have also looked at defining longitudinal trajectories of comorbidity burden. Lappenschaar et al^[44]^ used a Bayesian network to find associations between diseases and health risks to predict evolution of comorbidities over time (e.g., diabetic retinopathy and hypertension). Quiñones et al^[46]^ estimated ethnicity-specific comorbidity trajectories for white Americans, black Americans and Mexicans. These studies did not consider the association between longitudinal comorbidity burden and outcome, they focused on deriving trajectories that predict how quickly patients encounter new comorbidities, which is similarly important.

To our knowledge, this is the first study to report the prognostic impact of comorbidity burden evolution, as measured by CCI, in a natural/geographical population, and to consider the discrete contributions of cardiovascular versus noncardiovascular conditions.

Yet our analysis has several limitations. First, the SIR database relies on clinicians’ observations and entry of relevant codes into EHRs, which may be an incomplete or inaccurate representation of patients’ health. Most of the conditions in CCI, however, are recorded well in English primary care because they are part of the QOF pay-for-performance scheme. We observed a direct example of the effect of QOF in our dataset for renal disease. Particularly, prevalence in the Salford population raised from 1.5% in 2005 to 4.3% in 2006, and this is likely to be related to the introduction of renal disease in QOF during that year. Some cases will remain undiagnosed but this would be unusual given the serious nature of the CCI conditions, also dissent rates (i.e., refusal of assessment or treatment by the patient) in this context are low.^[47]^ Second, like most other investigators we considered CCI as a “rolling” measure, cumulating comorbidity burden until death. However, a limited number of the CCI disease categories (i.e., peptic ulcer or cancer) might be cured (a state not recorded in our EHR). Third, due to data-reuse restrictions, we did not have information about 2 CCI disease categories: sexual and mental health. We note, however, that HIV and dementia prevalence in our population is 4 in 1000^[48]^ and less than 1%, respectively.^[49]^ Given that the included data cover most of the disease burden of the population and the principal determinants of their outcomes we do not feel that the inclusion of sexual and mental health data would lead to substantially different findings. Fourth, general practices in Salford use 2 different EHRs, which can have a small influence over the data captured,^[50]^ but this is unlikely to be substantial for given the incentivized data capture for the CCI conditions. Finally, although our analyses focused only on the city of Salford, our cohort was composed of ∼280,000 patients and the data were collected from all 53 primary care practices in Salford. It is true that almost a third of neighborhoods in Salford are in the most deprived tenth of England. However, in terms of multimorbidity Salford is in the 61st centile. Therefore we expect that our results would be generalizable to other areas in England and UK.

Comorbidity burden is a dynamic process, with 1 in 10 patients in our study of British adults experiencing at least 1 change in comorbidity as measured by the CCI over a period of 5 years. Longitudinal models that include time-dependent CCI level and CCI change appear to be the most successful in capturing the effect of comorbidity burden on mortality and should be considered in survival analyses using EHR data—for research or for care quality management.

## Acknowledgments

The study was performed as part of the “Missed opportunities Detection” stream work at the Health eResearch Centre, which was approved by the Salford Integrated Record Board in July 2012. We thank the Salford Integrated Record Board for the help and support.

## Supplementary Material

Supplemental Digital Content

## References

[1] Uijen AA van de Lisdonk EH Multimorbidity in primary care: prevalence and trend over the last 20 years. *Eur J Gen Pract* 2008; 14 suppl 1:28–32. 18949641 10.1080/13814780802436093

[2] Koné Pefoyo AJ Bronskill SE Gruneir A The increasing burden and complexity of multimorbidity. *BMC Public Health* 2015; 15:415. 25903064 10.1186/s12889-015-1733-2 PMC4415224

[3] Wallace E Salisbury C Guthrie B Managing patients with multimorbidity in primary care. *BMJ* 2015; 350:h176. 25646760 10.1136/bmj.h176

[4] Boyd CM Fortin M Future of multimorbidity research: how should understanding of multimorbidity inform health system design? *Public Health Rev* 2011; 33:451–474.

[5] van Oostrom SH Picavet HSJ van Gelder BM Multimorbidity and comorbidity in the Dutch population—data from general practices. *BMC Public Health* 2012; 12:715. 22935268 10.1186/1471-2458-12-715 PMC3490727

[6] Barnett K Mercer SW Norbury M Epidemiology of multimorbidity and implications for health care, research, and medical education: a cross-sectional study. *Lancet* 2012; 380:37–43. 22579043 10.1016/S0140-6736(12)60240-2

[7] Fraccaro P Arguello Casteleiro M Ainsworth J Adoption of clinical decision support in multimorbidity: a systematic review. *JMIR Med Inf* 2015; 3:e4. 10.2196/medinform.3503 PMC4318680 25785897

[8] Yurkovich M Avina-Zubieta JA Thomas J A systematic review identifies valid comorbidity indices derived from administrative health data. *J Clin Epidemiol* 2015; 68:3–14. 25441702 10.1016/j.jclinepi.2014.09.010

[9] Clark DO Von Korff M Saunders K A chronic disease score with empirically derived weights. *Med Care* 1995; 33:783–795. 7637401 10.1097/00005650-199508000-00004

[10] Elixhauser A Steiner C Harris DR Comorbidity measures for use with administrative data. *Med Care* 1998; 36:8–27. 9431328 10.1097/00005650-199801000-00004

[11] Quan H Sundararajan V Halfon P Coding algorithms for defining comorbidities in ICD-9-CM and ICD-10 administrative data. *Med Care* 2005; 43:1130–1139. 16224307 10.1097/01.mlr.0000182534.19832.83

[12] Von Korff M Wagner EH Saunders K A chronic disease score from automated pharmacy data. *J Clin Epidemiol* 1992; 45:197–203. 1573438 10.1016/0895-4356(92)90016-g

[13] Charlson ME Pompei P Ales K A new method of classifying prognostic comorbidity in longitudinal studies: development and validation. *J Chronic Dis* 1987; 40:373–383. 3558716 10.1016/0021-9681(87)90171-8

[14] Wang CY Baldwin L-M Saver BG The contribution of longitudinal comorbidity measurements to survival analysis. *Med Care* 2009; 47:813–821. 19536031 10.1097/MLR.0b013e318197929c PMC2701975

[15] Zeng C Ellis JL Steiner JF Assessment of morbidity over time in predicting health outcomes. *Med Care* 2014; 52:S52–S59. 24561759 10.1097/MLR.0000000000000033 PMC8598243

[16] Strauss VY Jones PW Kadam UT Distinct trajectories of multimorbidity in primary care were identified using latent class growth analysis. *J Clin Epidemiol* 2014; 67:1163–1171. 25063556 10.1016/j.jclinepi.2014.06.003 PMC4165436

[17] Office for National Statistics (UK), Census 2011 (2011).

[18] NHS England, Read Codes (n.d.).

[19] Department for Communities and Local Government, English indices of deprivation 2015 (2015).

[20] Roland M Linking physicians’ pay to the quality of care—a major experiment in the United kingdom. *N Engl J Med* 2004; 351:1448–1454. 15459308 10.1056/NEJMhpr041294

[21] Sutton M Elder R Guthrie B Record rewards: the effects of targeted quality incentives on the recording of risk factors by primary care providers. *Health Econ* 2010; 19:1–13. 10.1002/hec.1440 19206084

[22] Taggar JS Coleman T Lewis S The impact of the Quality and Outcomes Framework (QOF) on the recording of smoking targets in primary care medical records: cross-sectional analyses from The Health Improvement Network (THIN) database. *BMC Public Health* 2012; 12:329. 22559290 10.1186/1471-2458-12-329 PMC4104830

[23] Olier I Springate DA Ashcroft DM Modelling conditions and health care processes in electronic health records: an application to severe mental illness with the clinical practice research datalink. *PLoS ONE* 2016; 11:e0146715. 26918439 10.1371/journal.pone.0146715 PMC4769302

[24] Kontopantelis E Reeves D Valderas JM Recorded quality of primary care for patients with diabetes in England before and after the introduction of a financial incentive scheme: a longitudinal observational study. *BMJ Qual Saf* 2013; 22:53–64. 10.1136/bmjqs-2012-001033 22918988

[25] Reyes C Estrada P Nogués X The impact of common co-morbidities (as measured using the Charlson index) on hip fracture risk in elderly men: a population-based cohort study. *Osteoporos Int* 2014; 25:1751–1758. 24676845 10.1007/s00198-014-2682-9

[26] Huang Y Gou R Diao Y Charlson comorbidity index helps predict the risk of mortality for patients with type 2 diabetic nephropathy. *J Zhejiang Univ Sci B* 2014; 15:58–66. 24390745 10.1631/jzus.B1300109 PMC3891119

[27] Ng ACC Chow V Yong ASC Prognostic impact of the Charlson Comorbidity Index on mortality following acute pulmonary embolism. *Respiration* 2013; 85:408–416. 23147354 10.1159/000342024

[28] Wu C-C Hsu T-W Chang C-M Age-adjusted charlson comorbidity index scores as predictor of survival in colorectal cancer patients who underwent surgical resection and chemoradiation. *Medicine (Baltimore)* 2015; 94:e43. 10.1097/MD.0000000000000431 PMC4602551 25590852

[29] Dias-Santos D Ferrone CR Zheng H The Charlson age comorbidity index predicts early mortality after surgery for pancreatic cancer. *Surgery* 2015; 157:881–887. 25704415 10.1016/j.surg.2014.12.006

[30] Erickson SR Cole E Kline-Rogers E The addition of the Charlson Comorbidity Index to the GRACE Risk Prediction Index improves prediction of outcomes in acute coronary syndrome. *Popul Health Manag* 2013; 17:54–59. 23965044 10.1089/pop.2012.0117

[31] Lu KJ Kearney LG Ord M Age adjusted Charlson Co-morbidity Index is an independent predictor of mortality over long-term follow-up in infective endocarditis. *Int J Cardiol* 2015; 168:5243–5248. 10.1016/j.ijcard.2013.08.023 23978361

[32] Rattanasompattikul M Feroze U Molnar MZ Charlson comorbidity score is a strong predictor of mortality in hemodialysis patients. *Int Urol Nephrol* 2012; 44:1813–1823. 22134841 10.1007/s11255-011-0085-9 PMC3595168

[33] Grossman R Mukherjee D Chang DC Preoperative Charlson Comorbidity Score predicts postoperative outcomes among older intracranial meningioma patients. *World Neurosurg* 2015; 75:279–285. 10.1016/j.wneu.2010.09.003 21492731

[34] Schmidt M Jacobsen JB Lash TL 25 year trends in first time hospitalisation for acute myocardial infarction, subsequent short and long term mortality, and the prognostic impact of sex and comorbidity: a Danish nationwide cohort study. *BMJ Br Med J* 2012; 344:e356. 22279115 10.1136/bmj.e356 PMC3266429

[35] Daskivich TJ Chamie K Kwan L Comorbidity and competing risks for mortality in men with prostate cancer. *Cancer* 2011; 117:4642–4650. 21480201 10.1002/cncr.26104

[36] Khan NF Perera R Harper S Adaptation and validation of the Charlson Index for Read/OXMIS coded databases. *BMC Fam Pract* 2010; 11:1. 20051110 10.1186/1471-2296-11-1 PMC2820468

[37] Cox DR Regression models and life-tables. *J R Stat Soc Ser B* 1972; 34:187–220.

[38] Akaike H A new look at the statistical model identification. *Autom Control IEEE Trans* 1974; 19:716–723.

[39] Quan H Li B Couris CM Updating and validating the Charlson comorbidity index and score for risk adjustment in hospital discharge abstracts using data from 6 countries. *Am J Epidemiol* 2011; 173:676–682. 21330339 10.1093/aje/kwq433

[40] Pencina MJ D’Agostino RB D’Agostino RB Evaluating the added predictive ability of a new marker: from area under the ROC curve to reclassification and beyond. *Stat Med* 2008; 27:157–172. 17569110 10.1002/sim.2929

[41] Pencina MJ Steyerberg EW D’Agostino RB Extensions of net reclassification improvement calculations to measure usefulness of new biomarkers. *Stat Med* 2011; 30:11–21. 21204120 10.1002/sim.4085 PMC3341973

[42] Wong J Taljaard M Forster AJ Addition of time-dependent covariates to a survival model significantly improved predictions for daily risk of hospital death. *J Eval Clin Pract* 2013; 19:351–357. 22409151 10.1111/j.1365-2753.2012.01832.x

[43] Townsend P Phillimore P Beattie A Health and Deprivation: Inequality and the North. Routledge: Croom Helm; 1988.

[44] Lappenschaar M Hommersom A Lucas PJF Multilevel temporal Bayesian networks can model longitudinal change in multimorbidity. *J Clin Epidemiol* 2015; 66:1405–1416. 10.1016/j.jclinepi.2013.06.018 24035172

[45] Aarts S van den Akker M Bosma H The effect of multimorbidity on health related functioning: temporary or persistent? Results from a longitudinal cohort study. *J Psychosom Res* 2015; 73:211–217. 10.1016/j.jpsychores.2012.05.014 22850262

[46] Quiñones AR Liang J Bennett JM How does the trajectory of multimorbidity vary across Black, White, and Mexican Americans in middle and old age? *J Gerontol Ser B Psychol Sci Soc Sci* 2011; 66B:739–749. 10.1093/geronb/gbr106 PMC3198247 21968384

[47] Doran T Kontopantelis E Fullwood C Exempting dissenting patients from pay for performance schemes: retrospective analysis of exception reporting in the UK Quality and Outcomes Framework. *BMJ* 2012; 344:e2405. 22511209 10.1136/bmj.e2405 PMC3328418

[48] Salford City Partnership, Sexual health in Salford (2012).

[49] Health and Social Care Information Centre, Quality and Outcomes Framework (QOF)—2014–15 (2015).

[50] Kontopantelis E Buchan I Reeves D Relationship between quality of care and choice of clinical computing system: retrospective analysis of family practice performance under the UK's quality and outcomes framework. *BMJ Open* 2013; 3:e003190. 10.1136/bmjopen-2013-003190 PMC3733310 23913774

