# Supplementary material for: Predicting mortality from change-over-time in the Charlson Comorbidity Index: A retrospective cohort study in a data-intensive UK health system
Source: Medicine (Baltimore). 2016 Oct 28;95(43):e4973. doi: 10.1097/MD.0000000000004973 (PMC5089087; doi:10.1097/MD.0000000000004973)
Supplement: Supplemental Digital Content [file medi-95-e4973-s001.doc]

**Supplementary Figure 1 and table 1:** Example of the process used to calculate build our datasets applied to a patient’s data. In supplementary table 1, the vertical blue dotted lines represent 12-month time windows. For each time window we reported in supplementary table 1 the information that we recorded in our dataset for this patient: age; gender; Charlson comorbidity index at baseline (i.e. to); Charlson comorbidity index; Charlson comorbidity index cumulative change (i.e. change between Charlson comorbidity index at baseline and current Charlson comorbidity index value); change between current Charlson comorbidity index value and the one at the previous time window; and a binary variable that tells if the patient died in the period of time between the current and the next time-window.


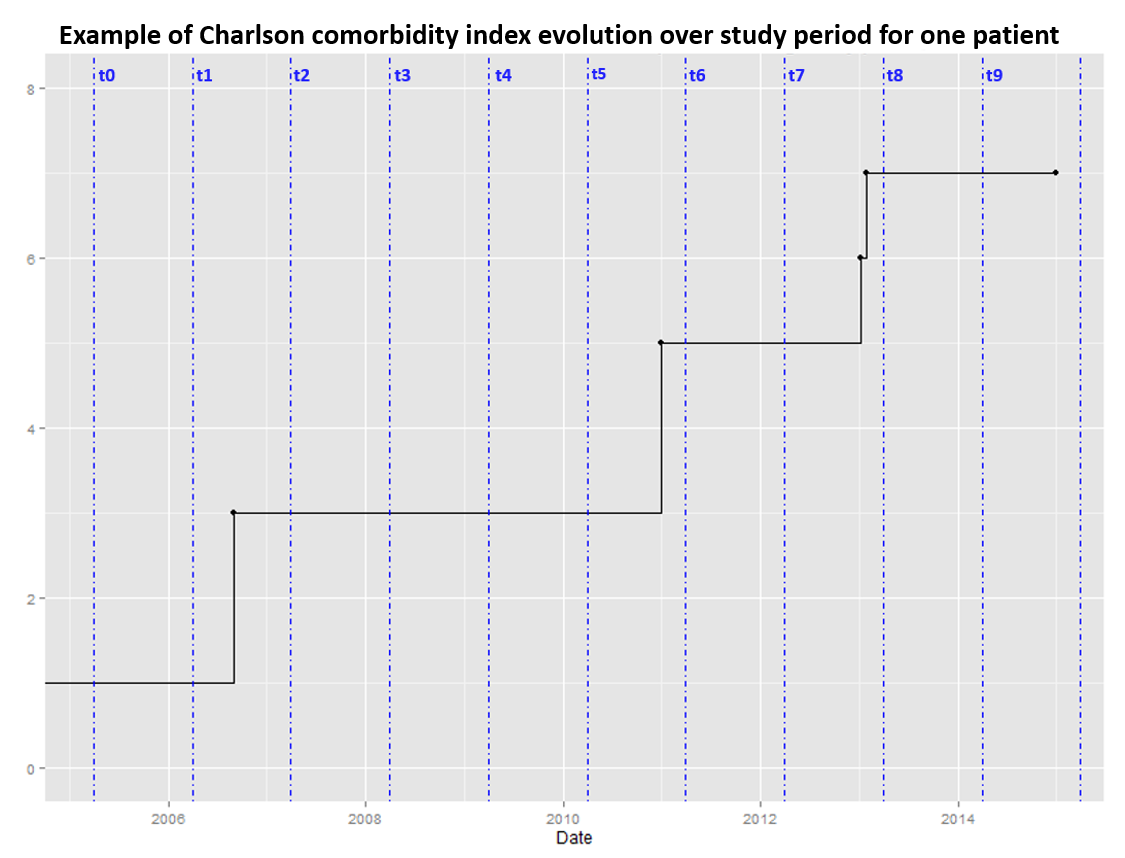


**Supplementary Figure 1 and table 1:** Example of the process used to calculate build our datasets applied to a patient’s data. In supplementary table 1, the vertical blue dotted lines represent 12-month time windows. For each time window we reported in supplementary table 1 the information that we recorded in our dataset for this patient: age; gender; Charlson comorbidity index at baseline (i.e. to); Charlson comorbidity index; Charlson comorbidity index cumulative change (i.e. change between Charlson comorbidity index at baseline and current Charlson comorbidity index value); change between current Charlson comorbidity index value and the one at the previous time window; and a binary variable that tells if the patient died in the period of time between the current and the next time-window.

| **Variable** | **t0** | **t1** | **t2** | **t3** | **t4** | **t5** | **t6** | **t7** | **t8** | **t9** |
| --- | --- | --- | --- | --- | --- | --- | --- | --- | --- | --- |
| Age | 58 | 59 | 60 | 61 | 62 | 63 | 64 | 65 | 66 | 67 |
| Gender | M | M | M | M | M | M | M | M | M | M |
| Charlson comorbidity index at baseline | 1 | 1 | 1 | 1 | 1 | 1 | 1 | 1 | 1 | 1 |
| Charlson comorbidity index | 1 | 1 | 3 | 3 | 3 | 3 | 5 | 5 | 7 | 7 |
| Charlson comorbidity index cumulative change | 0 | 0 | 2 | 2 | 2 | 2 | 4 | 4 | 6 | 6 |
| Charlson comorbidity index change | 0 | 0 | 2 | 0 | 0 | 0 | 2 | 0 | 2 | 0 |
| Death | NO | NO | NO | NO | NO | NO | NO | NO | NO | YES |


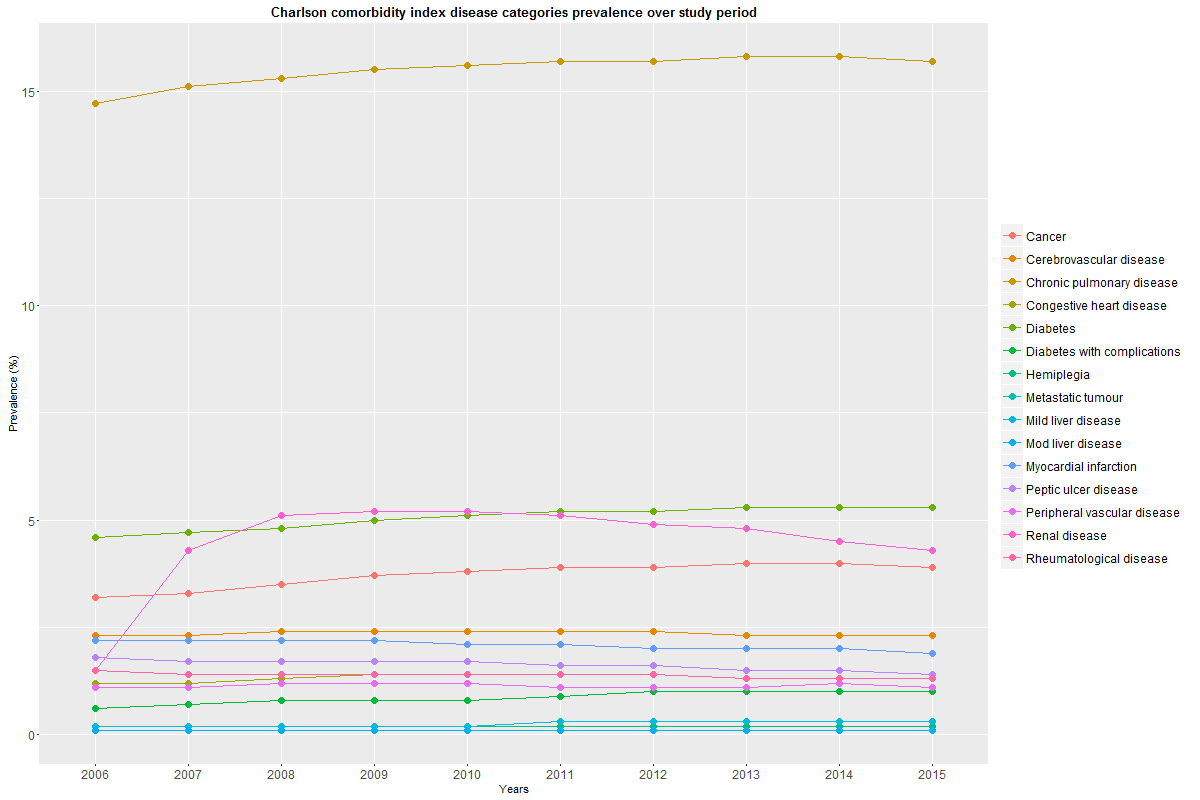


**Supplementary Figure 2:** Prevalence of the Chralson comorbidity index disease categories over the study period.


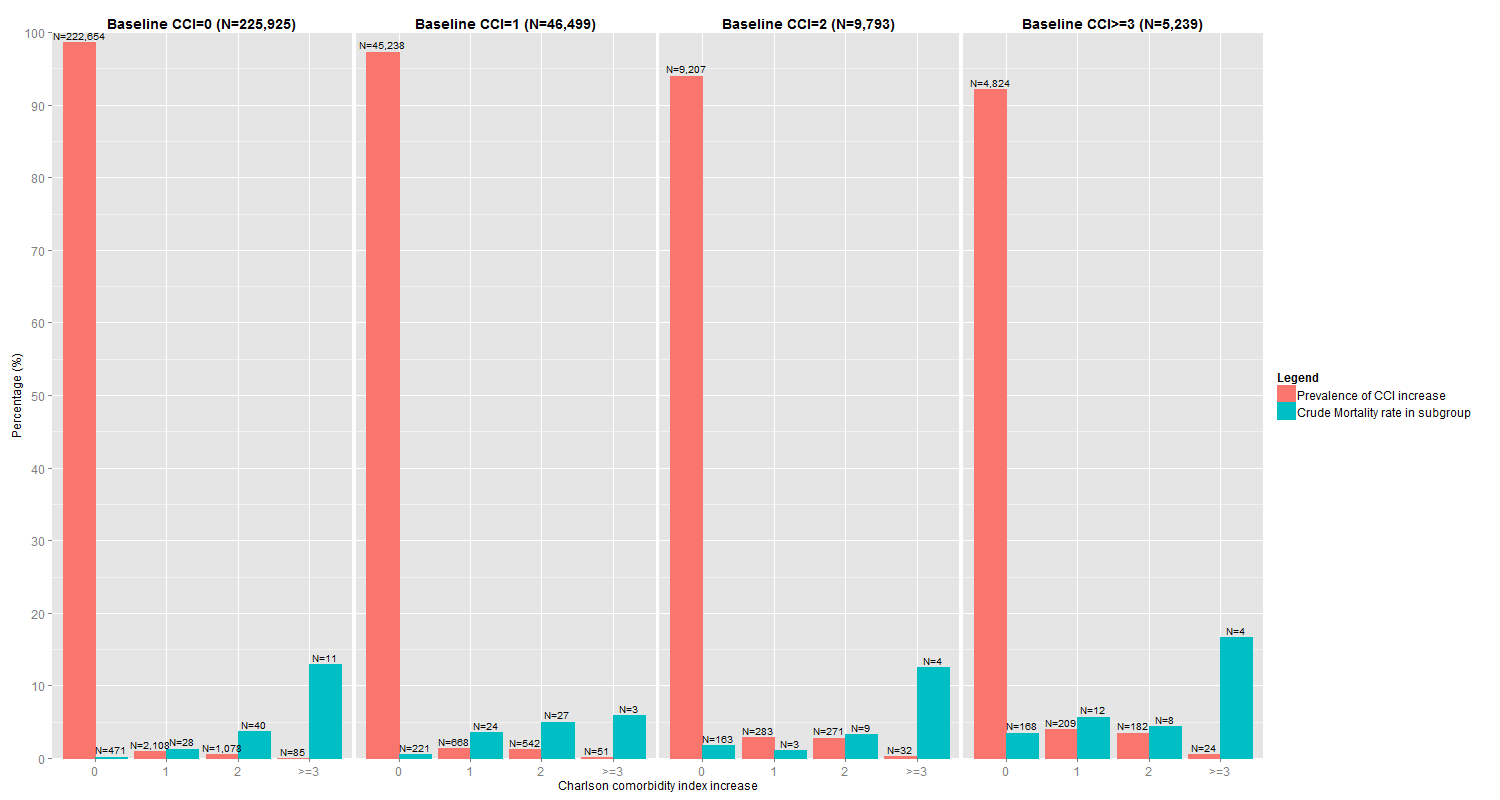


**Supplementary Figure 3:** Prevalence of Charlson comorbidity index increase (i.e. 0,1,2,>=3) in the SIR cohort after 1 year follow-up. Prevalence of increase is calculated on different subgroups on the basis of Charlson comorbidity index value at baseline (i.e. 0,1,2,>=3). For each subgroup, crude mortality is reported. Abbreviation: CCI, Charlson comorbidity index.


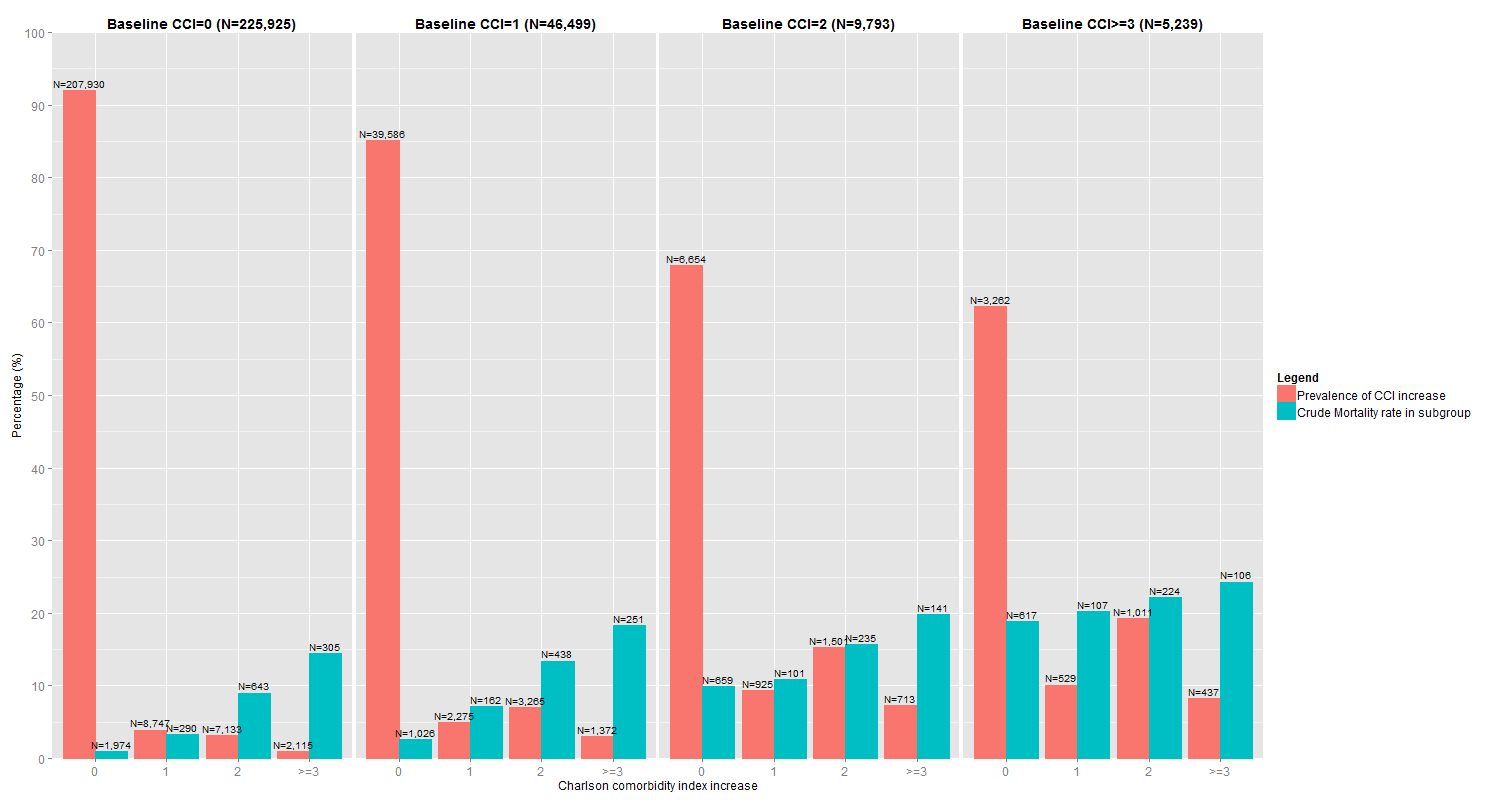


**Supplementary Figure 4:** Prevalence of Charlson comorbidity index increase (i.e. 0,1,2,>=3) in the SIR cohort after 5 years follow-up. Prevalence of increase is calculated on different subgroups on the basis of Charlson comorbidity index value at baseline (i.e. 0,1,2,>=3). For each subgroup, crude mortality is reported. Abbreviation: CCI, Charlson comorbidity index.


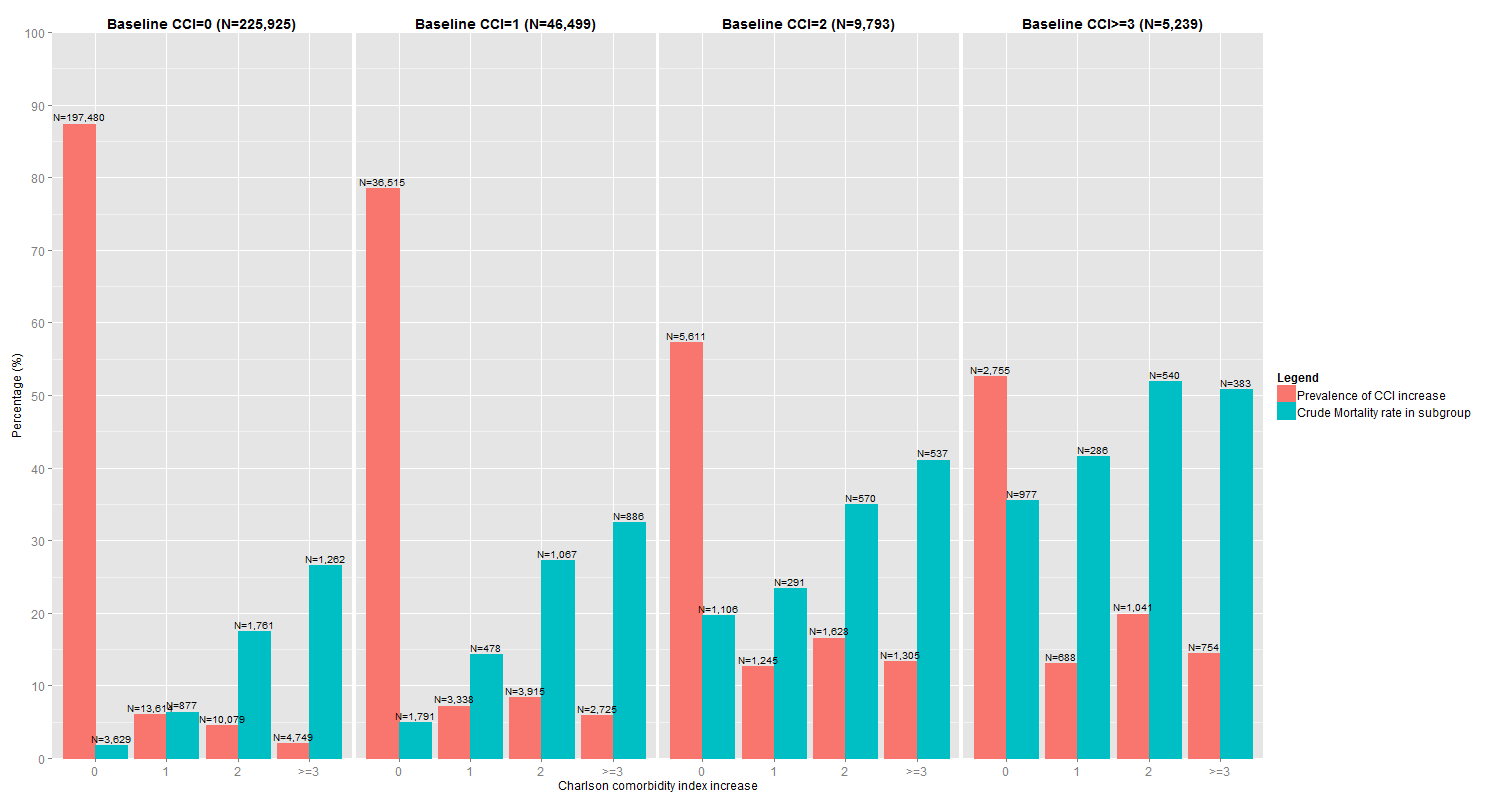


**Supplementary Figure 5:** Prevalence of Charlson comorbidity index increase (i.e. 0,1,2,>=3) in the SIR cohort over study period. Prevalence of increase is calculated on different subgroups on the basis of Charlson comorbidity index value at baseline (i.e. 0,1,2,>=3). For each subgroup, crude mortality is reported. Abbreviation: CCI, Charlson comorbidity index.


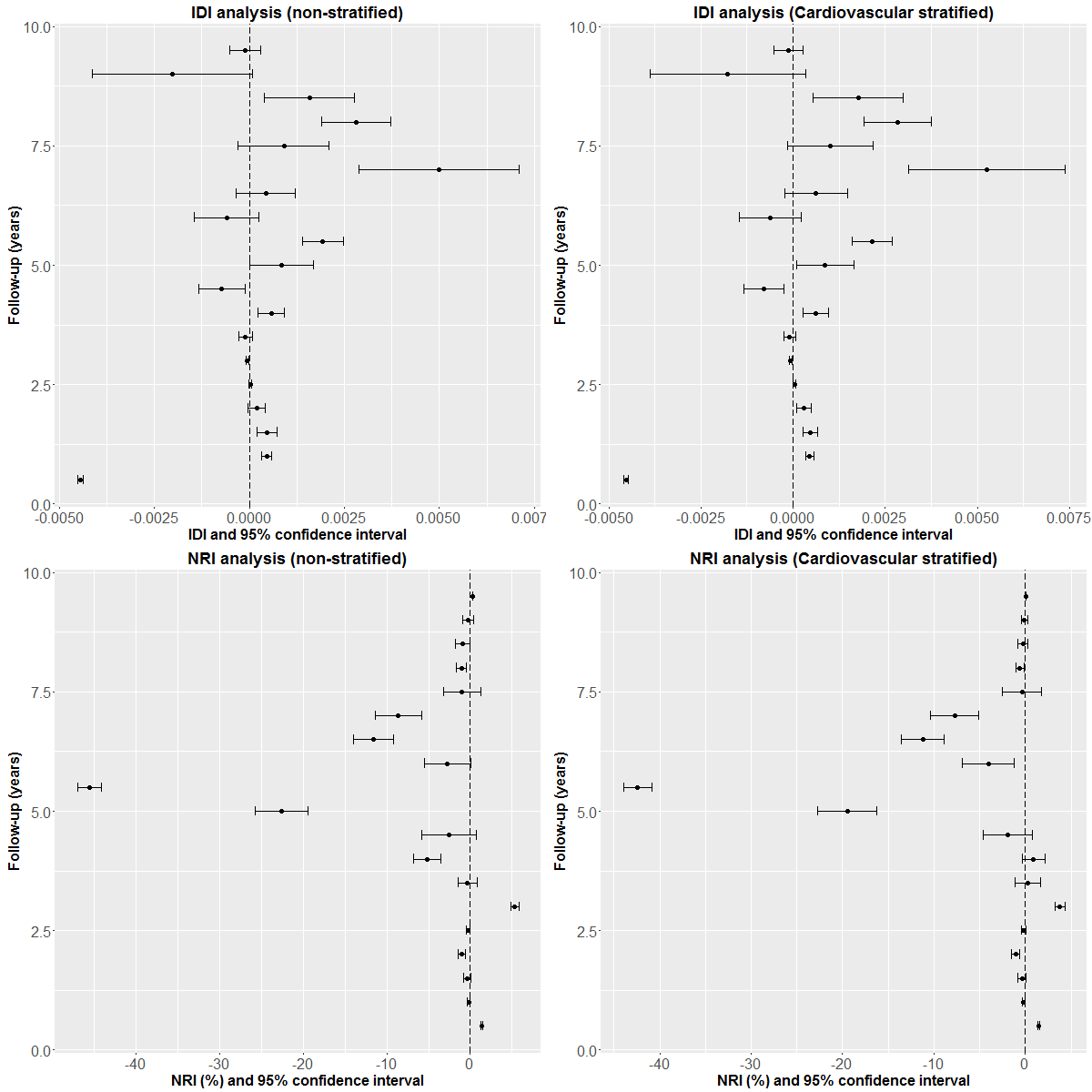


**Supplementary Figure 6:** Integrated Discrimination Index (IDI) and Net Reclassification Index (NRI) analysis to compare model 1 and model 5 for the 6-month time windows analysis. 95% confidence intervals are calculated from 100 bootstraps iterations.
